# Middle Ear Adenoma: Case Report and Discussion

**DOI:** 10.1155/2014/342125

**Published:** 2014-06-22

**Authors:** D. Isenring, T. F. Pezier, B. Vrugt, A. M. Huber

**Affiliations:** ^1^Department of Otorhinolaryngology, Head & Neck Surgery, University Hospital Zurich, Frauenklinikstraße 24, 8091 Zurich, Switzerland; ^2^Institute of Pathology, University Hospital Zurich, Rämistraße 100, 8091 Zurich, Switzerland

## Abstract

*Introduction*. Despite modern radiological workup, surgeons can still be surprised by intraoperative findings or by the pathologist's report. *Materials & Methods*. We describe the case of a 52-year-old male who was referred to our clinic with a single sided conductive hearing loss. He ultimately underwent middle ear exploration and excision of a middle ear tumour followed by second look and ossiculoplasty a year later. *Results*. Though preoperative CT and MRI scanning were suggestive of a congenital cholesteatoma, the pathologist's report diagnosed a middle ear adenoma. *Discussion*. Middle ear glandular tumors are extremely rare and, despite numerous histological techniques, continue to defy satisfactory classification. Most surgeons advocate surgical excision though evidence of the tumour's natural course and risk of recurrence is lacking.

## 1. Introduction

Middle ear glandular tumors are extremely rare and, despite numerous histological techniques, continue to defy satisfactory classification. Their cell line of origin, natural history, and progression if left untreated are unknown. Patients generally present with conductive hearing loss and most surgeons advocate surgical excision with or without immediate or delayed reconstruction of hearing.

## 2. Case Report

A 52-year-old patient was referred by a private ENT doctor to the University Hospital Zurich on suspicion of a right sided cholesteatoma. The patient described a loss of hearing and pressure sensation in the right ear which had been present for two months. The patient reported no pain, otorrhoea, dizziness, or systemic signs of infection. Past medical history was unremarkable. The patient took no medications and had no allergies.

**Figure 1:**
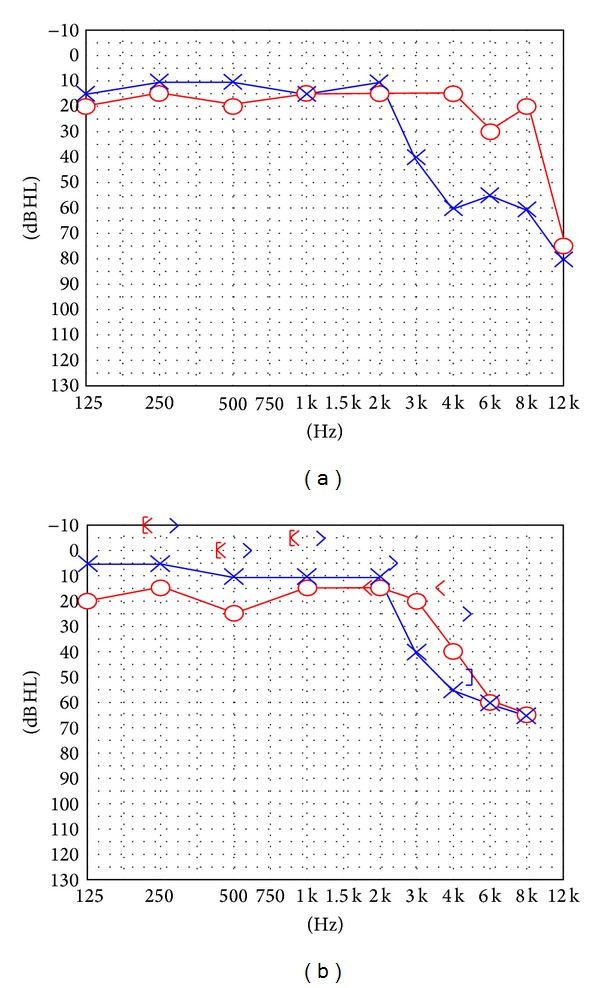
(a) Pure tone audiogram at presentation. (b) Pure tone audiogram 2 years following PORP.

**Figure 2:**
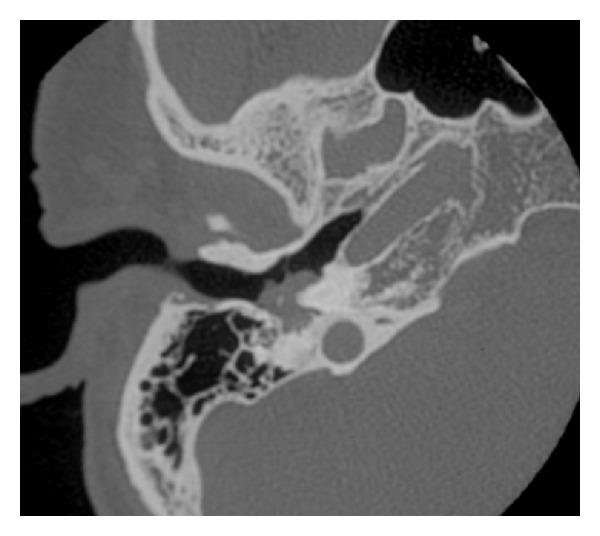
Computer tomogram at presentation.

Otoscopy showed an intact eardrum but with the suggestion of white mass behind the eardrum in the posterior superior/inferior quadrants. A pure tone audiogram (Figure 1) showed normal hearing in the right ear, with a high frequency sensorineural loss in the left. Temporal bone computer tomography showed a right sided middle ear soft tissue mass with possible erosion of the ossicles (Figure 2). A working diagnosis of a congenital cholesteatoma was made, and the patient was sent for MRI.

The MRI showed a 6 × 10 mm soft tissue mass, isointense on T1 and T2 sequences, with inhomogeneous gadolinium contrast uptake in the meso- and hypotympanum. Posterior to this was a 5 mm lesion, hyperintense signal on T2, without contrast uptake and without reduced apparent diffusion coefficient. All the inner ear structures were intact. These findings were felt to be more consistent with a glomus tumour than a cholesteatoma and the second lesion was felt to be artifact from a shine-through effect from mucosal swelling.

The patient underwent an explorative mastoidectomy, epitympanectomy, and tympanoplasty.

**Figure 3:**
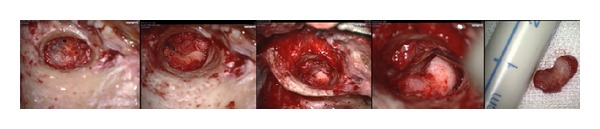
Intraoperative findings.

Intraoperatively (Figure 3), the whole middle ear was filled with the soft tissue mass apart from a small area superiorly/anteriorly. The chorda tympani had to be sacrificed along with an epitympanectomy to allow adequate visualization and excision of the mass.

**Figure 4:**
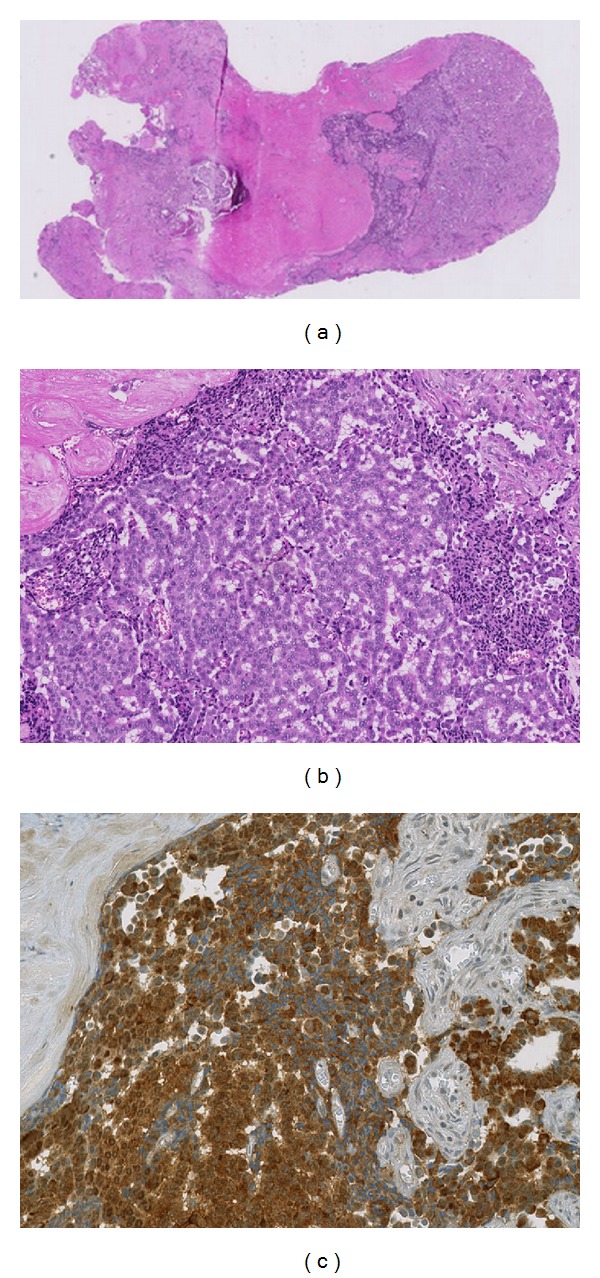
(a) Histological finding (overview), neuroendocrine adenoma (hematoxylin-eosin stain). (b) Histological finding (magnification), neuroendocrine adenoma (hematoxylin-eosin stain). (c) Histological finding (magnification), neuroendocrine differentiation (synaptophysin stain).

Pathological examination revealed a glandular and neuroendocrine tumor with areas of pleomorphic nuclei (Figures 4(a) and 4(b)). Immunohistochemically, the cells excessively expressed a marker protein for neuroendocrine cells (synaptophysin, Figure 4(c)) and partially chromogranin A. The proliferation rate was determined to be less than 1%. The diagnosis of a neuroendocrine differentiated middle ear adenoma was made, but the pathologist emphasized the need to rule out the possibility that this was a metastasis of an as yet unidentified primary.

A postoperative MRI scan performed 3 months later showed a discrete mucosal swelling in the hypotympanum with contrast uptake, possibly consistent with residual tumour. A further MRI scan 6 months later showed regression of this finding.

A full year after the initial excision, a second look was performed. No signs of persistence or recurrence were found, and a partial ossicular reconstruction prosthesis (PORP) was performed with good results (see Figure 1(b)).

## 3. Discussion

Middle ear glandular neoplasms are rare tumours thought to arise from the epithelial lining of the middle ear or a stromal precursor derived from the neural crest [1]. MEAs were first described by Hyams and Michaels in 1976 [1] and controversy exists as to what extent they represent a different entity from carcinoid tumours of the middle ear first described by Murphy et al. in 1980 [2]. Murphy felt that his case was better described as a carcinoid tumour because of ultrastructural evidence of a neuroendocrine differentiation. With only 94 published cases [3], current opinion leans towards classifying all these tumours as MEAs with a subset of neuroendocrine variants [4], as this appropriately implies their benign behavior. Some authors however argue that the appropriate terminology would be adenocarcinoid or amphicrine tumor in order to reflect its dual nature [5].

MEAs are grossly vascular tumors which are well circumscribed despite having no capsule. The most common presenting symptom is conductive hearing loss, though otalgia or a sensation of aural fullness is also commonly described. The mass tends to surround the ossicles without erosion, though in 8 other case reports, as with our case, bony erosion has been described. Facial paresis [4, 6] due to either compression or frank invasion [7] has also been reported. Only one case report describes a carcinoid syndrome with an MEA [8]. Middle ear carcinoid metastatic potential has also been described leading some to conclude that it should be considered a low-grade malignancy [7, 9, 10].

In 2009, Saliba and Evrard performed a thorough analysis of the literature and proposed a 3-tier classification according to immunohistochemistry (+/−) and metastasis (+/−). When both are negative, the tumor is described as an MEA. The most common type however shows positive immunohistochemistry and no metastasis and is described as a neuroendocrine adenoma of the middle ear. The rarest finding is of a tumor with both positive immunohistochemistry and metastasis/carcinoid syndrome and is described as a carcinoid tumor of the middle ear [3]. Of note, this paper mentions an average disease free interval of 53 months, but it is not clear how many patients had aggressive initial treatment, or indeed, how many actually had recurrence.

Pathologically MEAs are composed of exocrine and neuroendocrine cell types, sometimes with neuropeptides such as chromogranin, synaptophysin, serotonin, and pancreatic polypeptide [11]. They are predominantly composed of cuboidal-to-columnar cells with indistinct cytoplasmic borders. The nuclei tend to be round to oval with minimal pleomorphism [12]. The chromatin often shows a “salt and pepper” pattern consistent with neuroendocrine origin.

Treatment is surgical excision with or without the ossicles. Local recurrences rates as high as 12.7% are reported [3] and require the repeat of the operation. Adjuvant radiotherapy or chemotherapy as used for pulmonary or gastrointestinal carcinoid, even when the labyrinth is nonfunctioning, is discouraged [6].

## 4. Conclusions

Middle ear glandular neoplasms are rare tumors of the middle ear. Controversy still exists as to the most appropriate description and classification of these tumors and whether they should be subclassified in order to aid treatment and prognostication. Clinical suspicion of such a tumor mandates a thorough radiological workup, and patient fitness/consent aside, operative excision. This should be aggressive in order to keep down local recurrence rates and simultaneous or delayed reconstruction of the hearing may then be performed. Long term followup is recommended to rule out late recurrence and metastatic disease.

## 5. Summary

- Preoperative radiological workup does not always correlate with intraoperative findings or with the ultimate diagnosis. Clinicians should keep an open mind and be prepared to change their operative plan according to intraoperative findings.
- Middle ear glandular neoplasms are rare and controversy exists as to their appropriate classification.
- Currently, the classification by Saliba et al. is the most widespread and includes (in order of prevalence) neuroendocrine adenomas, adenomas, and carcinoid tumours according to immunohistochemical markers and metastasis.
- Adjuvant radiotherapy of chemotherapy is not recommended. Instead, a surgical second look can be performed, especially if interval imaging is suggestive of persistent disease.
